# Exploratory analysis of the correlation between precipitation and storms and malaria cases from national surveillance in Mozambique

**DOI:** 10.1186/s12936-026-05959-0

**Published:** 2026-06-11

**Authors:** Hengcheng Zhu, Lin Zhang, Euna Khan, Jaideep Srivastava, Mitchel Croal, Baltazar Candrinho, Mariana da Silva, Kelly M. Searle

**Affiliations:** 1 Division of Biostatistics and Data Health Science, University of Minnesota School of Public Health, Minneapolis, MN USA; 2 Department of Computer Science and Engineering, University of Minnesota College of Computer Science and Engineering, Minneapolis, MN USA; 3 National Malaria Control Program, Ministry of Health, Maputo, Mozambique; 4 Division of Epidemiology and Community Health, University of Minnesota School of Public Health, Minneapolis, MN USA

**Keywords:** Malaria, Climate, Precipitation

## Abstract

**Background:**

Climate change is impacting the seasonal weather trends. Mozambique is one country that has been experiencing significant impacts of climate change. These impacts include changing weather patterns and more frequent and intense storms. Mozambique also has the 5th highest malaria prevalence globally. These storms have all impacted the efficiency of the Mozambique National Malaria Control Program to achieve their goals. The goal of our study was to determine the appropriate temporal lag between high precipitation events and malaria risk in Mozambique. This knowledge is imperative to understand when and how to best respond the impacts of these events.

**Methods:**

Malaria monthly case count data at the district level were provided by the Mozambique National Malaria Control Program. Precipitation data were obtained from NASA GIS DISC Earth Data and were spatially aggregated to the district and temporally aggregated to the month to match the malaria surveillance data. We investigated the correlation between precipitation and malaria-related values at the district level each month accounting for spatio-temporal autocorrelation. We used Pearson correlation coefficients to quantify the correlations. We used Poisson generalized linear mixed models to determine the relative risk of malaria associated with precipitation nationally and for each of the three regions in Mozambique.

**Results:**

We found evidence of strong spatial variation in malaria cases, malaria hospitalizations, and malaria deaths. There was evidence of a clear temporal relationship between precipitation and malaria cases throughout Mozambique. Nationally, there was an increased relative risk for malaria at an 8-week temporal lag. However, this varied when stratified by region. In the Northern Region there was an increased relative risk for malaria between 6-week and 8-week temporal lags; in the Central region there was an increased relative risk for malaria between 10-week and 12-week temporal lags; and in the Southern region there was an increased relative risk for malaria between 10-week and 13-week temporal lags.

**Conclusion:**

With increasing storms and changing weather patterns due to climate change there is a need to understand the specifics of the association between elevated precipitation and malaria risk. We found important spatial heterogeneity in temporal lag times between precipitation events and malaria cases in Mozambique.

## Background

Malaria remains a major global public health issue with over 280 million cases and over 600,000 deaths [1]. This represents not only an increase in cases, but also deaths due to malaria, which is a change in the trends over two decades. One of the reasons for this shift has been the changing climate, which has resulted in increased frequency and severity of severe weather events [1]. There have been calls to action to address the highest burden areas and inequities in malaria control, however, these increases are troubling. There are multiple malaria prevention interventions available including, but not limited to: insecticide treated bednets; indoor residual spraying; intermittent preventative treatment during pregnancy; seasonal malaria chemoprevention; integrated community case management; and vaccination [1–4].

These tools typically are combined to form intervention packages by malaria control programs in endemic countries. While there have always been challenges in controlling malaria that have impeded efficacy of interventions (e.g. insecticide and drug resistance), recently there have been additional significant challenges for malaria control programs [2, 3].The COVID-19 pandemic made access to healthcare and interventions difficult in some environments and impossible in others [5]. Another major challenge is the impact that climate change has had on malaria transmission and risk [6–13].

Climate change is impacting the seasonal weather trends, particularly the typically predictable patterns in rain and temperature [14–17]. The seasonality of these trends are also associated with mosquito proliferation and risk of malaria infection [18–20]. In addition to these seasonal changes, there has been an increase in the frequency and intensity of severe weather events [14, 21–27]. These severe weather events include tropical storms, cyclones, floods, droughts, and heatwaves. While severe weather events have been increasing, they are still rare events epidemiologically, which makes it difficult to investigate their association with malaria risk directly. However, tracking precipitation patterns allows for continuous documentation and analysis of the impacts of both seasonal changes and severe weather events.

Mozambique is one country that has been experiencing significant impacts of climate change [24, 25, 28, 29]. These impacts include changing weather patterns and more frequent and intense storms [21, 25, 28, 29]. Mozambique also has the 5th highest malaria prevalence globally [3]. Current malaria prevention interventions implemented nationwide include ITN distributions though antenatal care centers and iCCM [3]. In the southern region of the country (Maputo, Inhambane, and Gaza Provinces) where the prevalence and incidence are lowest, there is a focus on local malaria elimination [30]. Additional interventions used in this area include mass ITN distributions and IRS. In the northern region of the country (Zambezia, Nampula, and Cabo Delgado) where the prevalence and incidence are highest, there is a focus on gaining control over transmission and preventing morbidity and mortality [30]. Additional interventions in this area include mass ITN distributions, school-based ITN distributions, and IRS [30].

Over the past 35 years, Mozambique has experienced over 75 declared natural disasters related to floods, droughts, tropical storms, and cyclones [31]. The most notable of these was Cyclone Idai in 2019, which was one of the worst tropical cyclones recorded in the Southern Hemisphere [32]. More recently there have been several impactful storms that have caused damage to each region of the country and resulted in severe flooding in the aftermath [28, 33, 34]. There have also been intermittent droughts and floods resulting from irregular seasonal rain patterns that have impacted the predictability of malaria cases [31].

These severe weather events have all impacted the efficiency of the Mozambique National Malaria Control Program (NMCP) to achieve their goals [21, 35, 36]. It is imperative to understand when and how to best respond the impacts of these events associated with a changing climate. Additionally, it is important to track these events to prepare in advance with additional prevention supplies (e.g. ITNs, IRS materials, testing and treatment materials, etc.).

There have been multiple studies that have investigated the temporal lags in precipitation and malaria risk in many locations. Most of these cover large geographic regions [37–41]. Currently, these studies show temporal lags around 8–10 weeks, however there is geographic variability [37–41]. The goal of our study was to determine the appropriate temporal lag between increased precipitation and malaria risk in Mozambique overall. We also aimed to investigate this by region of the country.

## Methods

### Malaria case data

Malaria case count data were provided by the Mozambique National Malaria Control Program (NMCP). These are surveillance data of malaria cases that present at health facilities (hospitals, health centers, and health posts) or to community health workers. These include both uncomplicated and severe malaria cases (hospitalizations) diagnosed by rapid diagnostic test or microscopy. The dataset also included malaria deaths. These are collected at each health facility and aggregated by month and transmitted to the District Health Office, these are then aggregated for the district and transmitted to the Provincial Health Office, then to the NMCP. We had access to monthly level malaria case counts per District from 2017 through 2022. These data also contained district level population counts per month to calculate malaria and incidence.

### Precipitation data

Precipitation data were obtained from NASA GIS DISC Earth Data. These were collected as gridded raster data at a spatial resolution of 0.1 × 0.1 degree and at a daily temporal resolution. We obtained data from 2016 through 2022 to allow for analysis of temporal lags in the association with malaria cases. We aggregated these to both weekly and monthly mean and total precipitation to the district level using a spatial join. This resulted in monthly mean and total precipitation per district from 2016 through 2022 throughout Mozambique and allowed us to track seasonal changes in precipitation and the impacts of high precipitation events like severe storms.

### Exploratory analyses

We mapped out the population distribution by year and district to determine if there were any temporal trends or changes in population over the analysis period. We then mapped out the malaria incidence per month by district to inspect the spatial and temporal distribution. We repeated these analyses for malaria hospitalizations and deaths.

We visually investigated the correlation between precipitation and malaria cases, malaria hospitalizations, and malaria deaths at the district level each month with attention to the spatial and temporal correlations. The goal of this analysis was to determine if there was an association between increases in precipitation and malaria outcomes and the appropriate temporal lag for the association between precipitation and malaria risk. This was done to assess both seasonal precipitation, and high precipitation events that accompany severe weather events. As there were lower numbers of malaria hospitalizations and deaths, we focused on malaria cases for the remainder of the analysis. We graphed the relationship between precipitation and malaria cases over time to estimate appropriate temporal lags for the entirety of Mozambique. We also investigated the temporally lagged effects of precipitation on malaria cases with temporal lags of 0-, 1-, and 2-months.

### Statistical and stratified analyses

We recognized spatial heterogeneity in the correlations between precipitation and malaria cases across Mozambique. We stratified the country into three regions (North, Central, South). To conduct a more granular analysis, we used weekly malaria case data, which excludes case data from community health workers, to investigate the association between temporal lags in precipitation and malaria risk. We used Poisson generalized linear mixed models (GLMs) to determine the relative risk of malaria associated with precipitation for each of the three regions in Mozambique. The GLM used 24 week’s precipitation values as the predictors to model the malaria cases, with the mean of all the precipitation as the reference value when calculating the relative risk. Specifically, let ${Y}_{i}$ denote the weekly malaria case values for the i th district, let ${X}_{lag-ij}$ denote the j th lagged weekly precipitation values for the i th district. We selected all districts belonging to the required regions (North, etc.) and fit the following Poisson GLM:

$$log(E(Y))={\beta}_{1}{X}_{lag-1}+{\beta}_{2}{X}_{lag-2}+...+{\beta}_{24}{X}_{lag-24}$$

where E(Y) denotes the expectation of weekly malaria case values, and ${\beta}_{j}$ denotes the estimated coefficient for the j th lagged weekly precipitation values from the model.

The relative risk (RR) values for the j th lagged week of the precipitation at the value of X are then predicted as $Exp[{\beta}_{j}(X-{X}_{ref})]$, where ${X}_{ref}$ is the reference value and we used the mean of all the precipitation data for the required regions.

## Results

**Fig. 1:**
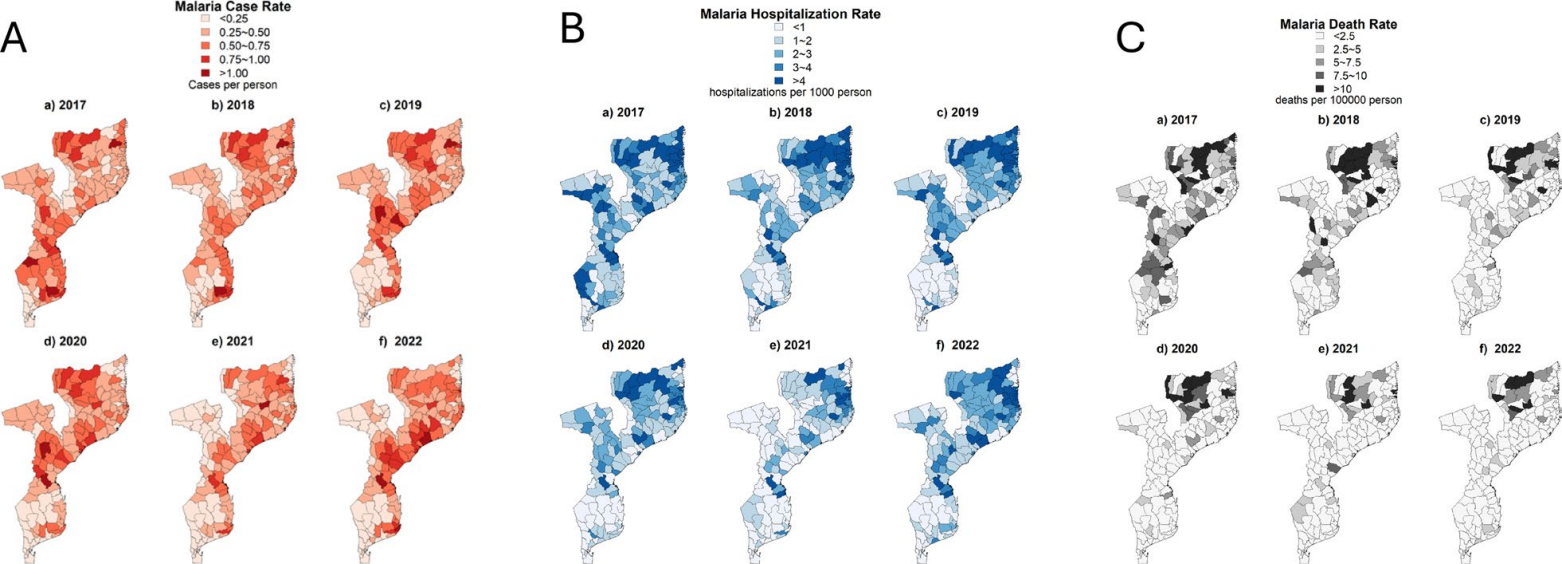
Distribution of **A** malaria cases, **B** malaria hospitalizations, and **C** malaria deaths by year (2017–2022)

We found evidence of strong spatial variation in malaria cases, malaria hospitalizations, and malaria deaths (Fig. 1). Malaria hospitalizations and deaths were more concentrated in northern districts, while malaria cases were distributed throughout the country (Fig. 1).

**Fig. 2:**
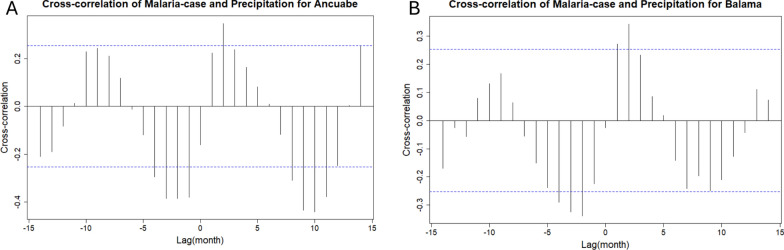
Cross-correlation of malaria cases and precipitation by monthly lag for a subset of two districts in Mozambique

**Fig. 3:**
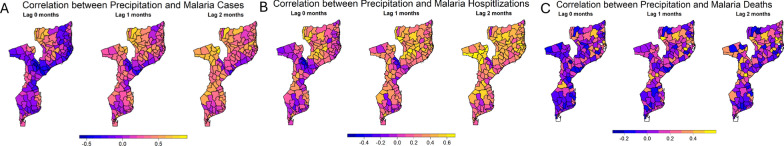
Spatial distribution of the correlation coefficient between precipitation and **A** malaria cases, **B** malaria hospitalizations, and **C** malaria deaths by temporal lags of lag0 (no lag), lag1 (1 month), and lag2 (2 months)

**Fig. 4:**
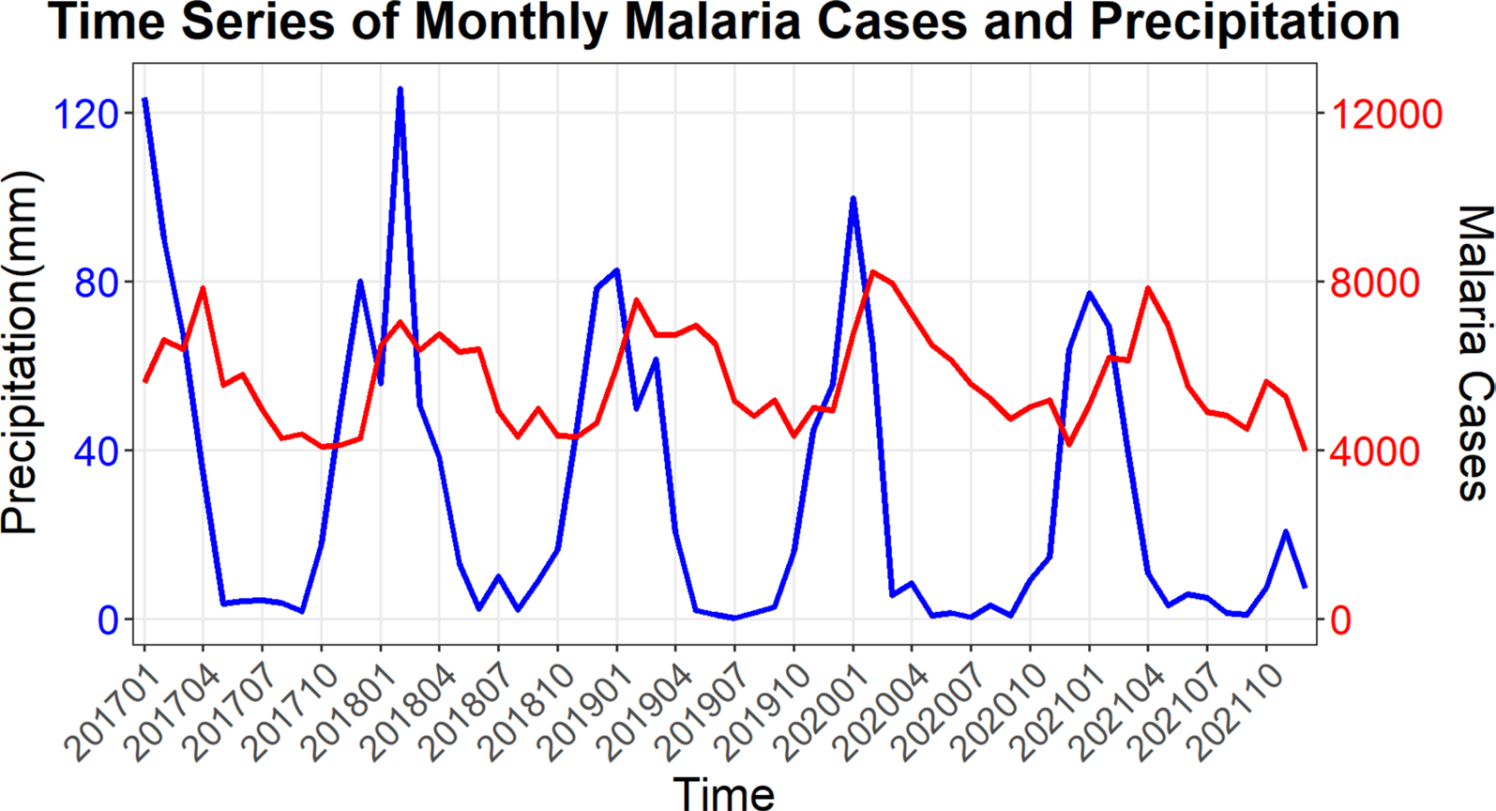
Time series between monthly malaria cases and monthly total precipitation averaged over all districts

Examining the cross-correlation between monthly precipitation and malaria cases across a subset of districts showed high levels of correlation at both 1-month and 2-month lags (Fig. 2). The spatial distribution of the correlation coefficients for malaria cases and malaria hospitalizations showed increased correlation across Mozambique for 1-month and 2-month lags (Fig. 3). The spatial distribution of the correlation coefficients for malaria deaths did not show substantial spatial heterogeneity and this did not change with different temporal lags (Fig. 3). When examining the time-series plots of precipitation and malaria cases (Fig. 4), there was evidence of a clear temporal relationship throughout Mozambique.

**Fig. 5:**
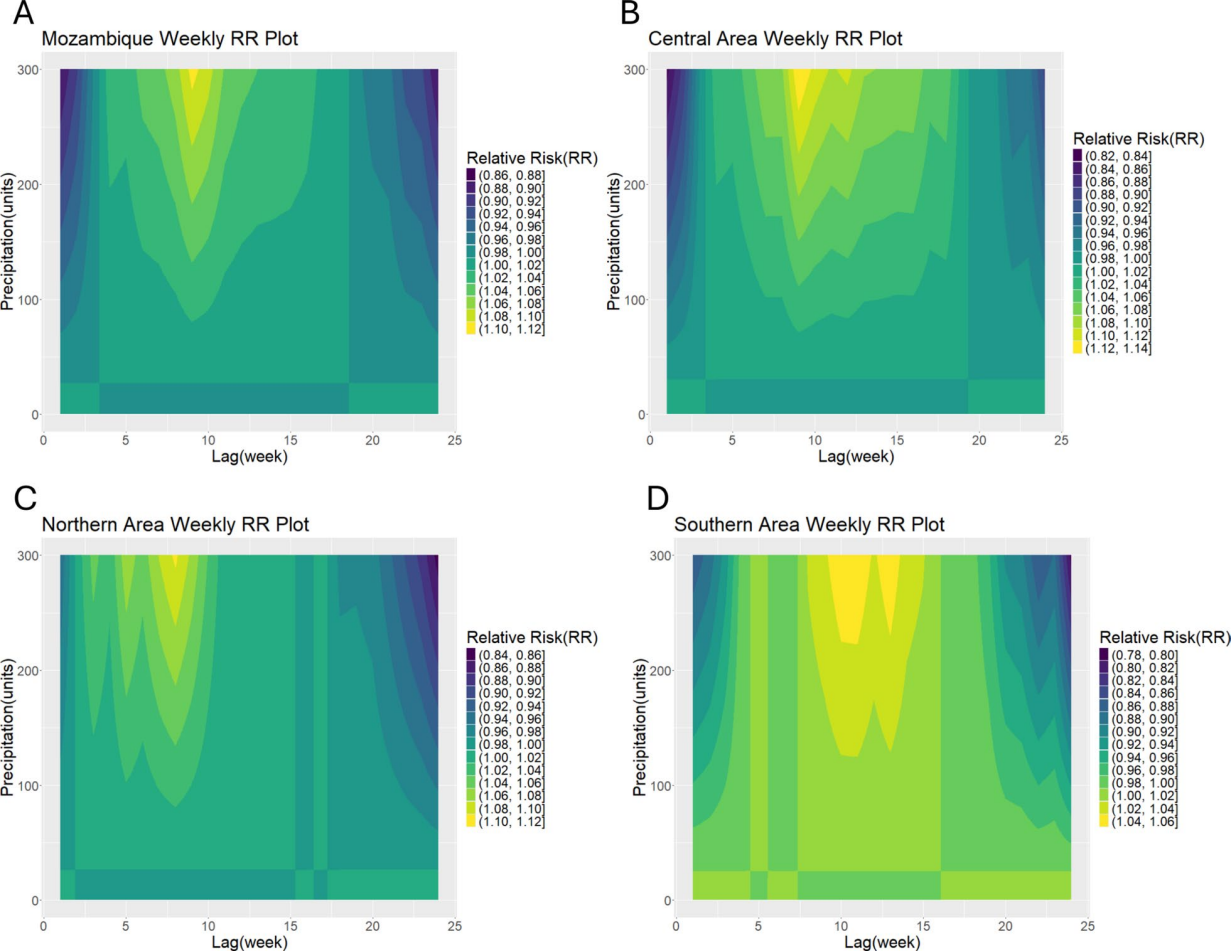
Heatmaps of the relative risk for malaria by weekly time lags

Heat maps of the association between precipitation and the relative risk of malaria from the model using weekly malaria case data as the outcome were created for all of Mozambique, and stratified by region (North, Central, and South) (Fig. 5). For the entire country, there is a clear increased relative risk for malaria at an 8-week temporal lag, which matches with the overall 2-month lag seen previously. However, this varied when stratified by region. In the Northern Region there was an increased relative risk for malaria between 6-week and 8-week temporal lags. In the Central region there was an increased relative risk for malaria between 10-week and 12-week temporal lags. In the Southern region there was an increased relative risk for malaria between 10-week and 13-week temporal lags.

## Discussion

In our study we found strong evidence of spatial and temporal correlation between increased precipitation and malaria cases. When we investigated the association between increased precipitation and malaria cases at the national level, our results were consistent with the current literature. These results indicated an approximate 8-week lag between elevated precipitation and increased malaria cases. However, when we stratified this by region of Mozambique, we found heterogeneity in these lag periods.

We found that in the Northern region of Mozambique, this lag is slightly shorter, between 6 and 8 weeks. In the Central region of Mozambique, we found the lag to be between 8 and 10 weeks. And in the Southern region of Mozambique the lag was 10–13 weeks. While all these values fall within ranges previously described in other locations, they represent important differences for both further modeling and decision making in Mozambique.

In Mozambique at the national level Armando et al. used a 1–2-month lag for precipitation, which is consistent with the 8-week lag that we found nationally [37, 39]. In a study in neighboring Zimbabwe by Gunda et al. a 4-week lag was used for the study area of interest [38]. Ghana Krefis et al. found a 9-week lag to be most appropriate for their study area [40]. These findings, along with others show that there is heterogeneity between countries and study areas in the appropriate lag time in the association between precipitation events and malaria risk.

We had several limitations to our study. We used both monthly and weekly malaria case data. The monthly case data included data from community health workers. However, the weekly case data did not include these. This underestimated our estimates in our weekly analyses, but this bias was non-differential across provinces and regions. Additionally, we attempted to include an indicator variable of the impact of severe storms and an interaction between severe storms and precipitation in our models. While severe storms are becoming more frequent, they are still rare events, and these models did not show any significance in the models and failed to converge when included as an interaction. Also, we only had six years of data for analysis. Longer time-series allow for more robust modeling of trends, specifically when investigating the associations between climate and malaria outcomes. In our study, malaria surveillance data were not available before 2016, but we plan to extend our analyses as more data become available.

Our study bolsters the current literature by investigating the specifics of the temporal lag in the association between precipitation and malaria risk in Mozambique. We have shown that at the national aggregate the lag time is consistent with other studies, while disaggregated to regions the lag times vary. These findings are important for informing modeling of climate and environment and malaria risk. Many studies use only aggregate data to determine lag times for models when there is geographic variability to also account for. Additionally, these findings are important programmatically for planning and responding to elevated precipitation events and severe storms. Knowledge of the specific lag times for different regions of Mozambique can be used to respond in a timely manner to changing seasonal trends and particularly in planning when to deliver interventions after a high precipitation event to have the maximum impact.

## Conclusion

With increasing storms and changing weather patterns due to climate change there is a need to understand the specifics of the association between elevated precipitation and malaria risk. We found important spatial heterogeneity in temporal lag times between precipitation events and malaria risk in Mozambique. These findings provide important information for further modeling and planning of the timing of interventions in response to storms and seasonal precipitation.

## Data Availability

The data used for this study were provided by the National Malaria Control Program in Mozambique. Requests for those data should be made to the Mozambique Ministry of Health.
